# Chronic Inflammation in the Context of Everyday Life: Dietary Changes as Mitigating Factors

**DOI:** 10.3390/ijerph17114135

**Published:** 2020-06-10

**Authors:** Denisa Margină, Anca Ungurianu, Carmen Purdel, Dimitris Tsoukalas, Evangelia Sarandi, Maria Thanasoula, Fotios Tekos, Robin Mesnage, Demetrios Kouretas, Aristidis Tsatsakis

**Affiliations:** 1 Department of Biochemistry, Faculty of Pharmacy, “Carol Davila” University of Medicine and Pharmacy, Traian Vuia 6, 020956 Bucharest, Romania; denisa.margina@umfcd.ro; 2 Department of Toxicology, Faculty of Pharmacy, “Carol Davila” University of Medicine and Pharmacy, Traian Vuia 6, 020956 Bucharest, Romania; carmen.purdel@umfcd.ro; 3 European Institute of Nutritional Medicine EINuM, 00198 Rome, Italy; dr.tsoukalas@gmail.com; 4 Metabolomic Medicine Clinic, Health Clinics for Autoimmune and Chronic Diseases, 10674 Athens, Greece; esarandi6@hotmail.com (E.S.); mariathanasoula84@gmail.com (M.T.); 5 Laboratory of Toxicology and Forensic Sciences, Medical School, University of Crete, 71003 Heraklion, Greece; 6 Department of Biochemistry-Biotechnology, School of Health Sciences, 41500 Larisa, Greece; fotis.tek@gmail.com (F.T.); dkouret@gmail.com (D.K.); 7 Gene Expression and Therapy Group, King’s College London, Faculty of Life Sciences and Medicine, Department of Medical and Molecular Genetics, 8th Floor, Tower Wing, Guy’s Hospital, Great Maze Pond, London SE1 9RT, UK; robin.mesnage@kcl.ac.uk; 8 Department Forensic Sciences and Toxicology, University of Crete, Faculty of Medicine, 71003 Heraklion, Greece

**Keywords:** diet, polyphenols, inflammation, real-life risk simulation, fasting, COVID-19

## Abstract

The lifestyle adopted by most people in Western societies has an important impact on the propensity to metabolic disorders (e.g., diabetes, cancer, cardiovascular disease, neurodegenerative diseases). This is often accompanied by chronic low-grade inflammation, driven by the activation of various molecular pathways such as STAT3 (signal transducer and activator of transcription 3), IKK (IκB kinase), MMP9 (matrix metallopeptidase 9), MAPK (mitogen-activated protein kinases), COX2 (cyclooxigenase 2), and NF-Kβ (nuclear factor kappa-light-chain-enhancer of activated B cells). Multiple intervention studies have demonstrated that lifestyle changes can lead to reduced inflammation and improved health. This can be linked to the concept of real-life risk simulation, since humans are continuously exposed to dietary factors in small doses and complex combinations (e.g., polyphenols, fibers, polyunsaturated fatty acids, etc.). Inflammation biomarkers improve in patients who consume a certain amount of fiber per day; some even losing weight. Fasting in combination with calorie restriction modulates molecular mechanisms such as m-TOR, FOXO, NRF2, AMPK, and sirtuins, ultimately leads to significantly reduced inflammatory marker levels, as well as improved metabolic markers. Moving toward healthier dietary habits at the individual level and in publicly-funded institutions, such as schools or hospitals, could help improving public health, reducing healthcare costs and improving community resilience to epidemics (such as COVID-19), which predominantly affects individuals with metabolic diseases.

## 1. Introduction

Chronic inflammation is a central process involved in a high number of metabolic disorders (e.g., obesity, metabolic syndrome, diabetes, dyslipidemia, etc.), including neurodegenerative (Alzheimer), malignant diseases, and autoimmune diseases. In most if not all chronic inflammatory conditions, there is an extensively failed resolution of inflammation with high influx of leukocytes, which in their effort to resolve inflammation stimulate the synthesis of pro-inflammatory molecules and establish a highly inflammatory micro-environment, leading to extensive fibrosis and tissue damage [1]. Chronic low-grade inflammation has been shown to either induce or aggravate metabolic disturbances, including insulin resistance and dyslipidemia, which contributes to the development of other complications [2]. There is accumulating evidence that, in the case of autoimmune diseases, when the immune system loses self-tolerance and attacks the body’s cells and tissues, metabolic disturbances are key contributors to disease progression. Results from the type 1 diabetes mellitus (T1DM) prediction and prevention studies on T1DM showed that metabolic disturbances preceded the seroconversion to positive autoantibodies by several months or years in type 1 diabetes mellitus [3,4].

Many chronic inflammatory diseases originate or have their development promoted by an unbalanced diet. Although the exact mechanism remains unclear, De Rosa et al. suggest that metabolic pressure, as a result of increased caloric intake, leads to an altered adipose tissue homeostasis. This results in the synthesis of adipokines and facilitates the overactivation of nutrient-sensing mechanisms, altering the balance between pro-inflammatory and regulatory T-cells, ultimately resulting in the loss of immunotolerance [5,6,7]. In addition, dietary components have the ability to influence the immune response through the modulation of gut bacteria metabolism, impacting the risk of developing chronic diseases either directly in the gastrointestinal tract, or in other more distant organs that impact general metabolism [8,9,10,11]. Recent studies have investigated long term exposure to low doses of chemical mixtures that can be a part of modern lifestyles, such as pesticides, food additives, or additives contained in food coating materials, proving that different disturbances appeared from minor biochemical disturbances. These early alterations are generally followed by oxidative stress induction and organ damage depending on the period of exposure [12,13,14,15,16,17]. Recently, it has been shown that long term exposure to stressors might also have a positive association with increased vulnerability of the population to the microbial and viral infections [18].

Metabolomics are an emerging biological field that allow for the identification and simultaneous measurement of a large number of small molecules called metabolites in biological matrixes. It has become the most accurate method to detect metabolic imbalances and is useful for prevention and early detection of diseases. Moreover, metabolomics have vast applications in clinical practice [19]. Targeted metabolomic analysis provides insights regarding the normal function of endogenous metabolism, dietary intake, microbiota, drug metabolism, and nutrient adequacy [20]. The challenge of chronic inflammatory diseases with respect to early diagnosis can be tackled with metabolomics through the identification of biomarkers that can discriminate high-risk populations. In a group of autoimmune patients, it was found that their fatty acid-based metabolic profile and lifestyle factors including physical activity and alcohol consumption were valuable predictive markers of autoimmune diseases [21].

Humans are exposed to a large number of substances from food, water, cosmetics, air, and so forth, each at low levels of exposure, and are able to induce cumulative/synergistic effects. Many studies have focused on the effects induced by administering a single substance at medium-high doses to laboratory animals. Recently, the concept of real-life risk simulation has emerged, since there is growing evidence that the effects of chemical mixtures at concentrations for which individual components failed to elicit have adverse effects when tested individually [14]. The concept of real-life risk simulation can also incorporate dietary interventions because, in our diets, we expose the human body to myriad substances in diverse doses [14,22,23].

The discovery of inflammation regulators opened a new window in therapeutics to clear low-grade chronic inflammation. A large number of physiological processes promote the physiological process of regulating inflammation. The development of such an approach targets the stimulation of endogenous processes that naturally occur during inflammation, which are hampered mainly by the lack of suitable human models and the heterogeneity of inflammatory disorders. Another limitation includes the lack of sensitive measurements able to capture the different stages of inflammation and metabolites [24,25,26,27,28].

The present paper aims to evaluate the impact diet might have on immune response, with special attention as to how lifestyle changes can help mitigate low-grade inflammation. real-life risk simulation (RLRS) concept. This analysis can be highly relevant in the context of the present viral spread of SARS-COV-2, since the inflammation is once again in the front line of an acute pathological response. Identifying strategies to modulate the immune response might prove useful for reducing the virus’s impact on the respiratory tract and thus diminishing its impact on each patient, as well as on the general medical system.

## 2. Food Frequency Questionnaires, Dietary Inflammation Index, and Metabolomics

The majority of studies that assess dietary habits, metabolism, and nutrient intake are based on food frequency questionnaires. However, food frequency questionnaires (FFQ) have several limitations, including inconsistent responses on food choices, mostly because answers depend on responders’ memory. Moreover, FFQ that are filled out by the responder instead of a trained healthcare professional only provide an overview of the macronutrients’ intake while not fully capturing the micronutrient status of the person [29]. Micronutrient deficiencies are common in both developing and affluent countries, affecting two billion people worldwide, according to the World Health Organization (WHO) [30]. The primary cause of micronutrient deficiencies or “hidden hunger” is poor dietary intake of micronutrients while other socioeconomic factors play an important role as well [31]. Several diet and nutrients assessment tools have been developed to evaluate inflammation status. The dietary inflammation index (DII) is based on literature data and aims to evaluate if a responder follows a pro- or anti-inflammatory diet. Since its development, there has been an increasing interest in DII, although other indexes are being developed with similar efficacy [32]. An important limitation of these indexes is the lack of causality and direct association to a person’s symptoms. Thus, the application of these indexes in clinical practice is hampered.

A novel empirical, close-ended, and self-administered questionnaire developed by the European Institute Of Nutritional Medicine provides an inflammation status score that captures the interaction between the autonomic nervous system and inflammation [33]. There is growing evidence that imbalances in the autonomic nervous system reflect local or systemic inflammation found in various diseases and that diet and lifestyle factors can act as regulators of sympathetic and parasympathetic activity [34,35,36,37]. Through a 23-question series, responders provide data on the presence/absence and status of inflammatory response in different body systems. Overall, the nutritional medicine exam (Numex) consists of 118 questions and assesses the nutritional deficiencies status in seven categories: inflammation, nutrition, perceived stress, oxidation, sugar metabolism, amino acids metabolism, and gut microbiome. Designed by medical doctors and nutritionists, the aim of this empirical test is to assist the individual and the healthcare professional to evaluate the overall inflammatory status based on autonomic nervous system changes and track its progression after targeted lifestyle changes. At a molecular level, metabolomics is the only method that can capture small, time-dependent fluctuations in the metabolism, thus indicating NCD-related metabolic imbalances [21]. Overall, traditional and well-established diet and nutrient assessment methods including FFQ and DII have provided valuable information on the role of specific foods on health and disease, as discussed in the present review. With the advent of advanced tools, metabolomics is complementary to the standard approach to provide tailor-made recommendations depending on an individual’s specific needs at a given time.

## 3. Changing Lifestyle as a Way to Mitigate Inflammation

Recent literature considers BMI cut-off values to not fully depict metabolic disturbances associated with obesity. The BMI is a mathematical approximation and does not reflect the percentage of total body fat between body fat and total body muscle or bone mass. As such, BMI does not reflect cardiometabolic risk. A more comprehensive classification describes four phenotypes for obese individuals: normal weight obese (NWO), metabolically obese normal weight (MONW), metabolically healthy obese (MHO), and metabolically unhealthy obese (MUO), or “at risk” obese with MS. This classification takes into account BMI, fat mass, and waist circumference, but also general biochemical parameters (e.g., fasting plasma glucose, total cholesterol, LDL, HDL, triglycerides) [38,39,40,41,42,43]. All four classes are characterized by impairments of different severity of inflammatory pathways [38,40].

**Figure 1:**
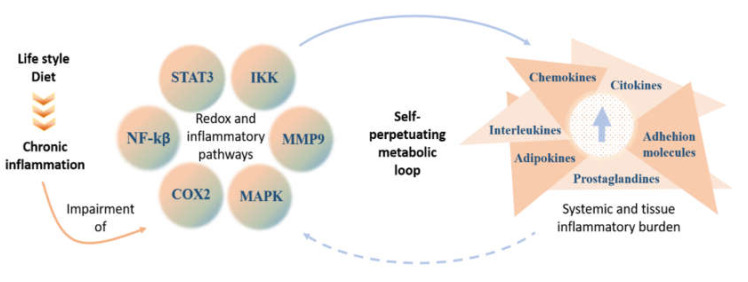
Impairment of metabolic pathways in lifestyle-associated low-grade chronic inflammation. STAT3 (signal transducer and activator of transcription 3), IKK (IκB kinase), MMP9 (matrix metallopeptidase 9), MAPK (mitogen-activated protein kinases), COX2 (cyclooxigenase 2), and NF-Kβ (nuclear factor kappa-light-chain-enhancer of activated B cells).

Lifestyle and nutrition are modifiable factors that interact with genetics in regulating chronic inflammation, leading to aforementioned complications. The changes in nutritional patterns in Western societies—caused by a high intake of fat and energy-dense, processed foods, as well as a low intake of fibers, fruits, and vegetables—are associated with a rising prevalence of asthma, allergies, and autoimmune diseases involving inflammatory mechanisms [44,45]. High fat diets determine, among other things: intestinal inflammation, favoring lipopolysaccharides (LPS) absorption from gram-negative gut bacteria, and increasing lipoperoxidation that induces insulin resistance and inflammation. Saturated fatty acids and LPS activate toll-like receptor 4 (TLR4) signaling pathways further contribute to promoting systemic inflammation and consequent metabolic disorders [46,47,48,49,50,51,52] (Figure 1).

Lifestyle- and diet-induced inflammation affects several cellular pathways, which stimulates the synthesis and secretion of various pro-inflammatory molecules. This ultimately maintains the low-grade inflammation state.

Interestingly, populations that consume a diet rich in fruits, vegetables, and fibers have lower incidences of inflammatory diseases compared to Western populations [53,54]. The Mediterranean diet—based on olive oil, fish, vegetables, and fruits, in addition to incorporating myriad beneficial phytochemicals—discourages cardiovascular diseases [55,56,57,58]. Sourcing food from organic agriculture could further improve the beneficial health effects of a Mediterranean diet, as suggested in a study comparing an organic and nonorganic Mediterranean diet on male patients with chronic kidney disease [59]. This was hypothesized to be due to a decreased exposure to pesticides, since animal studies have repeatedly found that exposure to pesticide mixtures can be a source of toxicity [16]. However, although most studies have found that organic food consumers are healthier, it is not clear whether health benefits can be attributed to a decreased exposure to synthetic pesticides [60].

Dietary changes that include specific metabolites can modulate gene expression via epigenetic modifications, such as DNA methylation or chromatin remodeling (e.g., histone acetylation or deacetylation). For example, a diet rich in folate and methionine can shape the host epigenome with a direct impact on molecular pathways associated with obesity-related inflammation. Moreover, global DNA hypermethylation in adipocytes derived from obese subjects is correlated with the expression of genes involved in proinflammatory interactions [61,62]. For example, hypermethylation at 1 kb upstream of the adiponectin gene’s promoter site was observed in adipocytes of obese mice fed a high-fat diet, but also in human adipocytes. DNA methyltransferase 1 (DNMT1) expression is correlated with the methylation of the adiponectin gene, resulting in decreased expression of adiponectin in obese mice and increased expression in healthy mice. Studies on human adipocytes show a correlation between DNMT1 expression and BMI, suggesting that obesity is a cause or cofactor of hypermethylation of adiponectin gene [63,64]. Another factor inducing epigenetic changes is ROS overproduction in expanded adipose tissue, influencing histone acetylation/deacetylation equilibrium, thus inducing NFκB activation [65,66,67].

On the other hand, nutrient restriction decreases AKT (protein kinase B) activity and stimulates FOXO (a forkhead box O transcription factor) activity, thus stimulating the expression of proteins involved in cell metabolism, autophagy, and stress-response, contributing to the resolution of inflammation [61,62]. Fasting regimens are correlated with increased insulin sensitivity, improvement of blood pressure, and inflammatory status, regardless if they are associated with weight reduction. For example, 15 days of intermittent fasting induced an increase of glucose uptake rates and a significant increase of anti-inflammatory adiponectin in lean young men (BMI of 25 kg/m^2^) without a significant decrease in body weight. These results were consistent with data from animal studies [68,69].

An important causal factor for low grade inflammation influenced mainly by lifestyle is the impairment of gut microbiota. Bacteroidetes and firmicutes constitute approximately 90% of the intestinal population, but the equilibrium is fundamentally changing with ageing and depending on diet composition. A decline of microbiota diversity occurs during ageing and obese individuals. Gut dysbiosis has been found in several inflammatory pathologies such as obesity, diabetes, cardiovascular, and neurodegenerative diseases. This can be connected to the induction of chronic low-grade inflammation since the gut microbiome is intimately connected to innate immune responses [70,71,72]. The relationship between gut microbiome and the host immune system are influenced by lifestyle interventions. For instance, secretory IgA levels increase after periodic fasting. This can be linked to changes in gut microbiome composition [14,73], with proteobacteria modulating the adaptive humoral local response.

Some studies showed that microbiota composition and diversity has a great impact on a population’s general health status. For example, when comparing the fecal microbiota of European and rural African children (Burkina Faso), a higher proportion of Prevotella and Xylanibacter (involved in the digestion of fibers and generation of short chain fatty acids (SCFAs)) was found in the latter group, which lacked European subjects. These observations could be correlated with the higher prevalence of inflammatory diseases in European populations compared to rural African ones [74,75].

Chronic exposure to environmental pollutants or food additives could also predispose one to chronic pathologies, which promotes inflammation [76]. Xenobiotics promotes chronic inflammation, which is thought to be the generation of lipotoxic conditions, i.e., in the development non-alcoholic fatty liver disease [77]. This can be mitigated by lifestyle interventions such as periodic fasting [78]. The exposure to xenobiotics such as heavy metals, pesticides, nanoparticles, polycyclic aromatic hydrocarbons, dioxins, furans, polychlorinated biphenyls, or non-caloric artificial sweeteners can also promote chronic inflammation by disturbing the gut microbiota [77,79,80].

Decreasing inflammatory burden is more important than ever during the COVID-19 pandemic. This can be accomplished through everyday actions (e.g., lifestyle, diet, smoking cessation, weight decrease, sport, etc.). There is a lot of information available in the scientific community regarding the risk of COVID-19 complications; even the likelihood of death is highly increased by some chronic diseases, mostly associated with an impaired inflammatory profile (e.g., obesity, type II diabetes, hypertension, chronic pulmonary disease, etc.) [81,82]. The literature data shows that people without comorbidities have a much lower risk of severe symptoms as a result of the SARS-COV-2 infection [83]. On the other hand, increased levels of inflammatory markers cytokines with pro-inflammatory outcomes constitute predictors of adverse outcome in COVID-19 patients [84]. Evidence proves that some dietary elements such as zinc or vitamin D might provide protective effects against viral load [84]. As such, this reduces the inflammatory burden through a healthy diet, associating (based on RLRS principles) several protective components (e.g., fiber, polyphenols, PUFAs, vitamins, etc.) that constantly increase our chance of being better protected against different immune challenges.

## 4. The Importance of Dietary Fiber for Reducing Inflammation

Fermentable dietary fiber are not enzymatically digested in the small intestine; they pass into the colon and are transformed by gut bacteria into SCFAs [85,86]. The systemic distribution and generation of SCFAs—acetate, propionate, and butyrate (the most abundant)—in the distal colon is important to inhibit inflammatory signals. Germ-free animal models were characterized by inflammatory flairs, due to the absence of tissue/blood SCFAs [87,88,89].

Butyrate is a representative member of SCFAs and has a high affinity for different G-protein-coupled receptors (GPCRs) found throughout the body: GPR41 is found in adipose tissues and immune cells and GPR43 is found in immune cells. However, GPR109A is present in colonic cells and GPR41 and GPR43 are activated by butyrate, which favors the production of peptide YY (PYY). This contributes to gastric emptying and intestinal transit inhibition, which thereby reduces appetite and promotes glucagon-like peptide 1 (GLP-1). These outcomes indirectly stimulate insulin secretion. GPR109A activates the inflammation-associated pathway in colonic macrophages and dendritic cells, inducing the differentiation of IL-10-producing T-cells and release of IL-18 from intestinal epithelial cells [85,90,91].

The presence of fiber in the diet is extremely important (Table 1) as it generates SCFAs and promotes the proliferation of commensal bacteria, which limits the access of pathogenic bacteria to the gut epithelium. Moreover, SCFAs favor epithelial mucus secretion that increases the protective effect on the intestinal surface and the proper maintenance of the barrier function [92,93]. SCFAs have anti-inflammatory effects that bind to the nuclear transcription factor PPARγ (peroxisome proliferator-activated receptor γ) and, consequently, inhibit the NF-kB pathway [94,95]. This ultimately lowers the expression of VCAM-1 (vascular adhesion molecule 1) and ICAM-1 (intracellular adhesion molecule 1), as well as the synthesis of TNFα, IL-6, and IFN-γ (interferon γ) [96].

The main physiological role of histones is to interact with DNA and stabilize its structure. When they are acetylated, this loosens the contact between histones and DNA, uncoiling the DNA structure that thus becomes transcriptionally active. The histone acetylation process is a result of the balance between the induction of histone acetyl transferases (HATs) and the inhibition of histone deacetylase (HDACs). The same acetylation process causes DNA to bind to transcription factors, such as STAT3 (signal transducer and activator of transcription 3), NF-Kβ (nuclear factor kappa-light-chain-enhancer of activated B cells, and FoxP3. Consequently, this regulates gene expression, including inflammation proteins [97,98]. SCFAs (with butyrate and acetate being the most and least effective, respectively) act as inhibitors for histone deacetylase (HDACs), thus contributing to the inhibition of the transcription for inflammatory proteins [99,100].

**Table 1:** Clinical studies regarding the effect of high fiber intake on inflammatory markers in obesity and associated pathology.

Design	Population	Dietary Intervention	Outcome	Reference
Randomized cross-over trial	50 Danish subjects with high risk of metabolic syndrome	two eight-week dietary intervention periods of whole grain intake (179 ± 50 g/day) and refined grain period (maximum 13 ± 10 g/day of whole grain), separated by a washout period of ≥6 weeks.	↓ body weight, serum inflammatory markers (IL-6, CRP)	[101]
Double-blind, randomized, placebo-controlled, crossover study	45 overweight adults with metabolic syndrome risk factors	galactooligosaccharide mixture intervention to increase dietary fiber content, with a 4-week wash-out period between interventions	↓ fecal calprotectin, CRP	[102]
Randomized controlled trial	143 individuals with metabolic syndrome	12 weeks of rye and whole wheat was compared with a diet containing the equivalent amount of refined cereal foods	no significant effects on the expression of inflammatory markers’ genes or insulin sensitivity	[103]
Randomized crossover study	19 adults with metabolic syndrome	4-week interventions diet enriched with arabinoxylan and resistant starch compared to a low-fiber Western-style diet	↓ fecal calprotectin, IL-23A and NF-κB	[104]
Crossover intervention study	25 hypercholesterolemic subjects	5-week intervention using high fiber (HF) and low fiber (LF) diet, separated by a 3-week washout.	↓ CRP and fibrinogen	[105]
Randomized controlled trial	68 overweight with prediabetes	12 weeks of 45 g/d of high-amylose maize (RS2) versus an isocaloric amount of amylopectin (control)	↓ TNF-α, no change in insulin sensitivity	[106]
Randomized controlled trial	166 subjects with features of metabolic syndrome	4-week using healthy diet (whole-grain products, berries, fruits and vegetables, rapeseed oil, three fish meals per week) compared to an average Nordic diet	Control diet: ↑ IL-1 Ra (versus healthy diet group)	[107]
Crossover study	10 healthy subjects	Subjects received either 910-calorie high-fat/high-carbohydrate meal or an American Heart Association (AHA) meal (fruit and fiber) during the first visit and the other meal during the second visit	↑ oxidative stress (plasma concentrations of TBARS, FFA, and LPS) and proinflammatory markers (TNFα, and IL-1β)	[108]
Randomized controlled trial	28 T2DM patients	Subjects received brown rice (n = 14) or white rice (n = 14) diet for 8 weeks	↓ CRP in brown rice group	[109]
Parallel design, dietary intervention trial	104 subjects with metabolic syndrome risk	Subjects received Healthy Diet (n = 44), a whole-grain-enriched diet (n = 42) or a control (n = 45) diet,	Healthy Diet group: ↓ E-selectin Healthy Diet and whole grain group: ↓ CRP	[110]
Randomized, double-blind, placebo-controlled, cross-over study	12 overweight and obese subjects	Subjects received 20 g/day of inulin-propionate ester, a high-fermentable fiber control (inulin) and a low-fermentable fiber control (cellulose) for 42 days	IPE: ↓ IL-8 levels (versus cellulose) Inulin: no effect on the inflammatory markers	[111]
Crossover clinical study	18 subjects at low-to-moderate cardiometabolic risk	Subjects received breakfast rich in saturated fatty acids (SFA), the other in unsaturated fatty acids (unSFA) and fiber for 4 weeks	SFA: ↑ IL-1β unSFA: ↓ IL-6	[112]
Interventional diet study	21 overweight/obese children	Subjects were placed on a regimen of ad libitum, high-fiber, low-fat diet, and daily exercise regimen for 2 weeks	↓ IL-6, IL-8, TNFα, PAI-1, resistin, amylin, leptin, insulin, and IL-1Ra ↑ adiponectin	[113]
Randomized, placebo-controlled study	31 hemodialysis patients	Patients received either resistant starch or placebo supplementation for 4 weeks	↓ IL-6 and TBARS	[114]
Randomized controlled clinical trial	55 women with T2DM	Subjects received a daily supplement of 10 g resistant dextrin or a similar amount of maltodextrin for 8 weeks	↓ IL-6, TNF-α and MDA ↑ Insulin sensitivity	[115]
Randomized cross-over double-blind placebo-controlled trial	17 obese knee osteoarthritis patients	Patients received freeze-dried strawberries or placebo for 2 periods of 12 weeks with 2 weeks of wash-out	↓ TNF-α and 4-HNE	[116]
Randomized study	59 T2DM patients	Patients received metformin, acarbose, and either a high fiber or a low fiber diet intervention for 8 weeks	Low fiber group: ↓ IL-18	[117]
Crossover study	33 healthy, middle-aged adults	Patients received either high or low in in wholegrain intervention for 6-week periods, separated by a 4-week washout.	Whole grain: a slight decrease of IL-10 and CRP	[118]
Observational study	8 subjects with impaired fasting glucose	subjects received (1) high-fiber formula; (2) high-monounsaturated fatty acid formula or (3) control formula	High fiber group: ↓ NF-κB in PBMCs	[119]
Randomized controlled clinical trial	60 females with T2DM	Patients received 10 g/d resistant starch or placebo for 8 weeks, respectively	↓TNF-α, no effect on IL-6 or CRP	[120]
Crossover clinical trial	80 overweight subjects	Subjects received two isocaloric breakfast interventions -one rich in saturated fat and one in unsaturated fatty acids and fibers for 4 weeks with a 2-weeks washout.	Fiber group: ↓ IF-γ and TNF-α	[112]
Observational study	49 T2DM females	Patients received either 10 g/day inulin or maltodextrin/day for 8 weeks	Inulin: ↓CRP, TNF-α and LPS	[121]
Randomized controlled clinical trial	52 overweight/obese women with T2DM	Patients received either 10 g/d of oligofructose-enriched inulin or maltodextrin (control) for 8 weeks	oligofructose-enriched-Inulin: ↓ CRP, TNF-α and LPS	[122]
Randomized crossover clinical trial	44 overweight/obese girls 8–15 years old	Subjects received either whole-grain or control for 2 periods of 6 weeks with 4-week washout period	Whole grain: ↓ CRP, ICAM-1 and leptin	[123]

IL-6, IL-1β, IL-6, IL-8, IL-18, IL-10—interleukin 6, 1β, 6, 8, 18, 10; IL-1 Ra—interleukin 1 receptor agonist; CRP—C reactive protein; NF-κB—nuclear factor kappa B; TNF-α—tumor necrosis factor α; TBARS—thiobarbituric acid reactive substances; FFA—free fatty acids; LPS—lipopolysaccharide; MDA—malondialdehyde; 4-HNE—4-hydroxynonenal; IF-γ—interferon γ; ICAM-1—intercellular adhesion molecule 1.

## 5. Anti-Inflammatory Effects of Fasting

Fasting is a process that has been known for thousands of years. It was quite frequent in ancient times, because access to food was difficult and as a result, individuals were obliged to survive without food until it was available again [124].

Fasting is a survival mechanism in both animals [125,126] and humans, especially in countries where food conservation is not widespread [127]. In the rest of the world, fasting has been employed either due to religious convictions or in wellness centers. Since 1960, one of the methods used to address morbid obesity and related diseases has been the “zero calorie diet”, thus translating into clinical practice the scientific data generated by centuries of fasting.

Fasting can be divided into three broad categories:

- Periodic fasting, which lasts from 2 days to a few weeks;
- Intermittent fasting, which lasts from 16 to 20 h and can be done daily or every second day or twice a week, and
- Fasting-mimicking diet, the diet that mimics fasting to achieve its beneficial effects, in which restriction of calories and specific foods is necessary (e.g., fat) [128,129,130].

There is a lot of research that shows the beneficial effects of fasting on health and also on different pathological conditions. Fasting increased lifespan in prokaryotic organisms such as yeast S. Cerevisiae and nematode C. Elegans [128,131,132,133,134], but also on animal models that performed fasting for long periods (e.g., the royal penguin) [135]. Other models have shown better brain function [136,137], increased lifespan and longevity [138,139,140], and improved maintenance of muscle mass after fasting [141].

Studies on animal models reveal the beneficial effect fasting has on cancer as a complementary disease management strategy in concert with drug treatments [142,143,144,145]. Studies on animal models show an improvement in neurodegenerative diseases after fasting, while other studies prove that intermittent fasting diets boost the levels of antioxidant defense, neurotrophic factors (BDNFS, H-70 and FGF2), proteins involved in adaptive response (HSP-70 and GRP-78), and reduce pro-inflammatory cytokines levels (TNFa, IL-1β, and IL-6) [146,147,148,149,150].

It has been found that intermittent fasting can prevent and reverse all aspects of metabolic syndrome in rodents: body fat, inflammation, and blood pressure are reduced; insulin sensitivity is increased; and the functional capacity of the neuromuscular and cardiovascular systems are improved [151,152,153]. An intermittent fasting diet has also been found to improve hyper-glycaemia in diabetic rodent models [154] and in myocardial infarction models, as the heart is protected from ischemic damage by this type of regimen [155]. Elevated leptin levels usually predict a pre-inflammatory condition, while adiponectin and ghrelin may suppress inflammation and increase insulin sensitivity [150,156]. Fasting can reverse every major abnormality caused by metabolic syndrome, by increasing insulin and leptin sensitivity, suppressing inflammation and stimulating autophagy [157,158].

There are several studies that show an increased use of fat and ketone bodies for energy [159,160], as well as an increase of growth hormone and glucagon secretion [161,162,163], with a decrease in blood sugar, insulin, and IGF-1 levels. After intermittent fasting, total fat, abdominal fat, and blood pressure are decreased, while glucose metabolism is improved in obese individuals [69,164,165,166,167]. In addition, periodic fasting significantly changes the composition of the human gut microbiota [73]. Finally, studies of utmost importance show the effect of intermittent fasting mainly in the fight against cancer as a supplement along with the classic treatment, with promising results [168,169]. Below, we analyze the effects of the interrelation between fasting and inflammation as well as the relevant molecular mechanisms.

### 5.1. Molecular Mechanism of Fasting

Fasting not only results in weight loss but it is a survival mechanism that impacts many metabolic pathways [128,161,170]. Fasting’s many benefits are related to the regulation of key molecular pathways. Initially, during fasting, the downregulation of insulin-like growth-factor-1 (IGF-1) and mammalian target of rapamycin (mTOR) occur. These pathways are upregulated in the presence of food excess as they sense nutrients and therefore activate anabolic metabolism. When there is a lack of food for several hours, catabolic processes are activated.

Aging appears modulated by changes in the insulin-like growth-factor-1 receptor signaling system, as longevity is enhanced by a decrease in IGF-1 signaling [171,172,173]. The IGF-1 signal induces mTOR activation. Reduced mTOR activity is related to extended lifespan in different organisms [174], as mTOR induces activation of FoxO proteins. FoxO proteins are transported to the nucleus and activate genes associated with autophagy [175], which emphasizes the link between autophagy and FoxO proteins.

When the AMP/ATP ratio is high, the AMPK path is activated [176]; this results in increased energy production and reduced ATP utilization. In addition, the mitochondrial biogenesis and mitophagy repair and replace damaged mitochondria. As a result, the cells have “younger” and more efficient mitochondria. In addition to the aforementioned condition, activation of this path has been associated with increased lifespan in various studies in both C. Elegans and Drosophila melanogaster [177,178]. In mammals, fasting does not appear to affect AMPK activation, but further studies are needed to be able to draw surer conclusions [179].

Like AMPK, sirtuins are associated with life [180] and autophagy [181]. Some sirtuins are found in the cytoplasm (SIRT2), some (SIRT1) in the nucleus having DNA repair action, and others in mitochondria. In general, sirtuins are associated with mitochondrial biogenesis and mitophagy for damaged mitochondria, thereby enhancing mitochondrial cells without problems and are thus more efficient in energy production [132,182]. SIRT1 is modulated by NAD^+^ level and is increased in energy depletion states (such as fasting or exercise) for which NAD^+^ is a sensor, which contributes to the reduction of inflammation through NF-Kβ down-regulation and related transcription factors [132,182,183].

A study on rats showed that inflammation decreased with fasting [184]. Other work has shown NF-Kβ inhibition and the modulation of Nrf2, sirtuins, SOD2, and increased lifespan [185,186,187,188]. In a 2017 study, intermittent fasting appears to significantly reduce corticosterone (CORT), interleukin 6 (IL-6), and tumor necrosis factor-alpha (TNF-α) levels [189].

**Figure 2:**
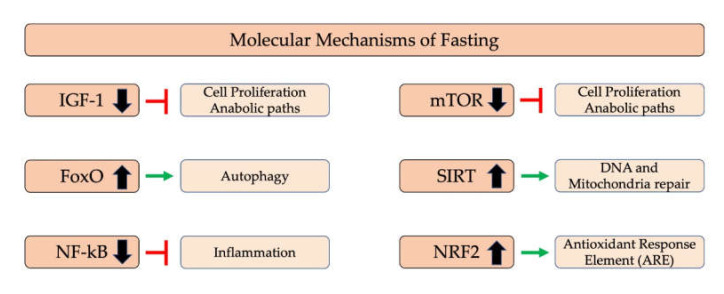
Molecular mechanisms of fasting. IGF-1, insulin-like growth factor 1; FoxO, forkhead box Proteins; NF-Kβ, nuclear factor-Kβ; mTOR, mammalian target of rapamycin; SIRT, sirtuins; NRF2, nuclear factor erythroid 2-related factor 2.

Nrf2 plays a key role in oxidative stress and toxicity; the right balance in ROS levels is very important so that mitochondrial and all other pathways can function properly. The absence of ROS, however, does not activate Nrf2, which in turn does not activate ARE (antioxidant response). Thus, a critical amount of ROS (“Hormesis hypothesis”) is necessary for the upregulation of ARE, which allows cells and mitochondria to be able to deal with oxidative stress and different kinds of toxins [190], which consequently increases their lifespan [191] (Figure 2).

### 5.2. Effects of Fasting on Humans

During the last decades, many studies have been conducted on the effect of fasting on several markers related to metabolism. Most of them determine the effect of fasting on weight. However, there are several studies that identify changes in lipid and carbohydrate metabolism as well as key hormones that affect the above (e.g., insulin). Recently, some studies have focused on fasting’s effect on inflammatory markers such as TNF-α, interleukins, CRP, and BDNF, as well as the hormones adiponectin and leptin.

The largest study on fasting’s effects is an observational study including 1422 subjects that describes metabolic changes after a 4- to 21-day fasting period [159,170]. All the participants fasted according to the Buchinger Wilhelmi fasting guidelines, which include a daily caloric intake of 200–250 kcal together with a variety of lifestyle changes (e.g., dietary advices, physical exercise). A beneficial modulating effect of fasting was observed on blood lipids, glucoregulation, and altogether general health-related blood parameters. Additionally, it was associated with a reduction in weight, abdominal circumference, and blood pressure. In another study, which used the same fasting guidelines, improved metabolic markers were observed after periodic fasting, including a decrease in blood glucose levels associated with changes in gut microbiome composition [73]. In this study, the analysis of the gut microbiome after 10 days of periodic fasting showed that fasting caused a decrease in the abundance of bacteria known to degrade dietary polysaccharides such as Lachnospiraceae and Ruminococcaceae, concomitant to an increase in Bacteroidetes and proteobacteria known to use host-derived energy substrates.

A study of eight healthy non-obese men discovered that in 15 days of fasting every other day, with 20 h fasting on fasting days, adiponectin increased and leptin decreased, while no changes in IL-6 or TNF-α were observed. Protocol allowed them to maintain normal exercise but also to consume food in order to keep their weight stable [69].

Redman et al., in a two-year study of 34 people who followed a reduced calorie intake diet (15%) observed a reduction in leptin. These individuals lost an average of 8.7 kg while the control group gained an average of 1.8 kg in the same period [192].

Another study including eight women and two men, all overweight with asthma, showed that fasting every other day and reducing calories to less than 20% of their normal intake on the days of fasting, for eight weeks, resulted in a reduction in TNF-α and BDNF, but no change in CRP. In this study, patients lost 8% of their initial weight during the study. Asthma symptoms also improved as well as some indicators of oxidative stress (8-protein carbonyls, isoprostane, nitrotyrosine, and 4-hydroxynonenal adducts) [193].

In 2013, another study [194] found that 12 weeks of reduced calorie intake every other day resulted in reduced CRP levels, increased adiponectin levels, and reduced leptin levels in 30 adults who were either overweight or normal weight. On fasting days, they consumed only 25% of the calories they normally consumed each day. In addition, their weight was significantly reduced by 3.6 ± 0.7 kg and the coronary heart disease risk was improved as the concentration of TG reduced.

Another study [195] found reduced high sensitivity-C-reactive protein (hs-CRP) of 27 women with polycystic ovary syndrome (PCOS) during the Ramadan period in Iran and in which participants aged 18 to 40 years old with an average of age 27.5, followed everyday 16.5 h fasting, isocaloric diet, for 29 days.

An important study involving 34 men (resistance-trained) who fasted every day 16 h, followed an isocaloric diet for eight weeks, and consumed 100% of their energy needs in the 8-h eating window, showed that there was an increase in adiponectin and a reduction of leptin of IL-6 and IL-1β [196].

Faris’ study on 50 healthy volunteers (21 men and 29 women) fasting for 14–15 h each day for 21 days, showed a reduction in IL-6, IL-1β, TNF-α, total leukocytes, granulocytes, lymphocytes, and monocytes [197].

Another study [198] involving 42 patients aged 20 to 50 years old with nonalcoholic fatty liver disease, following Ramadan fasting (every day 16-h fasting for 29 days), showed a reduction in IL-6 and hs-CRP, compared to 41 volunteers who did not fast. These and other studies are presented in detail in Table 2.

Collectively, an increasing number of studies show that fasting has numerous health benefits and could be used to prevent or manage the development of cardiometabolic disorders, metabolic diseases, and immune diseases. Although extended periods of fasting can be challenging without medical advices, recent studies also showed that time-restricted eating can be practiced safely as a routine. For instance, a recent study showed that 10 h of time-restricted eating for 12 weeks improved cardiometabolic health for patients with metabolic syndrome [199].

**Table 2:** Recent reports concerning fasting and inflammation.

Intervention Duration and Type of Fasting	Population	Comparison Group or Condition	Effects	Reference
15 days: alternate day fasting (20 h fasting intervals)	8 males non-obese adults	None	↑ adiponectin, ↓ leptin ↓ glucose, NS insulin, NS IL-6, NS TNF-α	[69]
29 days: every day 16.5 h fasting, isocaloric diet	27 females, polycystic ovary syndrome (PCOS)	None	NS insulin, NS LDL, NS HDL, NS TG, ↓hs-CRP (*p* = 0,072), ↑GSH, NS MDA, NS TAC	[195]
22 days: no caloric intake every other day (36 h fasting intervals)	8 females, 8 males, non-obese adults	None	NS glucose, ↓ insulin,↓ weight	[167]
8weeks: every day 16 h fasting, isocaloric diet	34 resistance-trained males	2 groups time-restricted feeding (TRF) or normal diet group (ND).	NS Weight, ↓Fat mass, ↑Adiponectin, ↓ Leptin, ↓IL-6, ↓IL-1β, NS insulin, NS T3, NS Glucose, NS Cholesterol, NS Cortisol, NS HDL, NS LDL, TG↓,	[196]
1 day: water only (28 h fasting interval)	20 females, 10 males Healthy adults	None	↓ glucose, ↓ insulin, ↓ weight, ↑ LDL ↑ HDL, ↓ TG, NS CRP, NS adiponectin	[200]
29 days: every day 16 h fasting	83 patients with NAFLD	42 fasted, 41 control, comparison between groups	↓Weight, ↓BMI, NS BF, ↓Glucose, ↓insulin, ↓IL-6, ↓hs-CRP	[198]
20 weeks: 1 day per week fast OR 5-day consecutive fasts every 5 weeks (400–600 kcals on fasting days)	31 females, 23 males Overweight or obese diabetics	1200–1500 kcal weight loss diet	↓ weight, NS glucose, NS insulin, NS LDL, NS HDL	[201]
21 days: every day 14–15 h fasting	Fifty (21 men and 29 women) healthy volunteers	7 days before fasting vs. 21 days after fasting	↓Weight, ↓BMI, ↓BF%, ↓SBP, ↓DPB, ↓IL-6, ↓IL-1β, ↓TNF-α, ↓Total ↓leukocytes, ↓Granulosytes, ↓Lymphocytes, ↓Monocytes	[197]
8 weeks: <20% of usual intake on alternate days. Ad libitum diet on non-fasting days.	8 females, 2 males Overweight adults with asthma	None	↓ TNF-α, ↓ BDNF, ↓ weight, NS glucose, NS insulin, NS LDL, ↑ HDL, ↓ TGs, NS CRP, NS leptin, ↓ Protein Carbonyls, ↓ Nitrotyrosine, ↓ 8-isoprostane	[193]
2 years caloric restriction	34 CR (15%) and 19 control for 2 years	comparison between groups	↓Weight, ↓BMI, ↓ BF, ↓ Leptin ns, Insulin NS, ↓ 2,3-dinor-iPF(2α)-III	[192]
8 weeks: weight loss diet with alternate day modified fasting (~25% of total energy needs)	12 females, 8 males Obese adults	None	↓ weight, ↓ LDL, NS HDL, ↓ TGs	[165]
6 months: 25% energy restriction 2 days per week	107 females Young, overweight or obese adults	25% energy restriction 7 days per week	NS CRP, NS adiponectin, NS leptin NS BDNF NS glucose, ↓ insulin NS glucose, ↓ insulin	[166]
12 weeks: 25% of energy needs alternating with ad libitum intake	39 females, 2 males Obese adults	Control group	↓ weight, NS CRP, NS glucose NS insulin, NS LDL, NS HDL, NS TG	[202]
6±3 years caloric restriction	18 CR and 18 control	comparison between groups	↓Weight, ↓BMI, ↓ BF, ↓ LDL, ↓HDL, ↓TG, ↓ Cholesterol, ↓insulin, ↓glucose, ↓hs-CRP, ↓SBP, ↓DBP	[203]
6 weeks: 25–30% energy needs on Sat. Mon, Wed; ad libitum other days	15 females Overweight or obese	None	↓ weight, NS LDL, NS HDL, NS TG	[204]
12 weeks: 25% energy restriction 2 consecutive days per week	37 females Overweight or obese women	25% energy restriction all days of week	NS adiponectin, NS leptin, NS IL-6, NS TNF-α, NS glucose, NS HbA1c, ↓ insulin, NS LDL NS HDL, NS TG, NS weight	[205]
10 weeks intermittent fasting (IF)-calorie restriction (CR) regimen (with or without liquid meals)	54 were randomized to either the IFCR-liquid (IFCR-L) or IFCR-food based (IFCR-F) diet	comparison between 3rd and 10rd week in each group	In IFCR-L: ↓Leptin, ↓IL-6, ↓TNF-α, NS CRP, ↓IGF-1, In IFCR-F: ↓Leptin, NS IL-6, NS TNF-α, NS CRP, NS IGF-1,	[206]
12 weeks: weight loss diet with alternate day modified fasting (~25% of energy needs)	22 females, 8 males Normal and overweight adults	Control group	↓ CRP, ↑ adiponectin, ↓ leptin NS LDL, NS HDL, ↓ TG, ↓ weight	[194]

NS—non-significant; hs-CRP—highly sensitive CRP; LDL—low density lipoproteins; HDL—high density lipoproteins; GSH—glutathione; TAC—total antioxidant capacity; T3—triiodotironine; TG—triglycerides; BMI—body mass index; BF—body fat; SBP—systolic blood pressure; DBP—diastolic blood pressure; 2,3-dinor-iPF(2α)-III –2,3-dinor-8-iso Prostaglandin F 2α; HbA1C—glycated hemoglobin; IGF-1—insulin growth factor 1.

## 6. Conclusions

**Figure 3:**
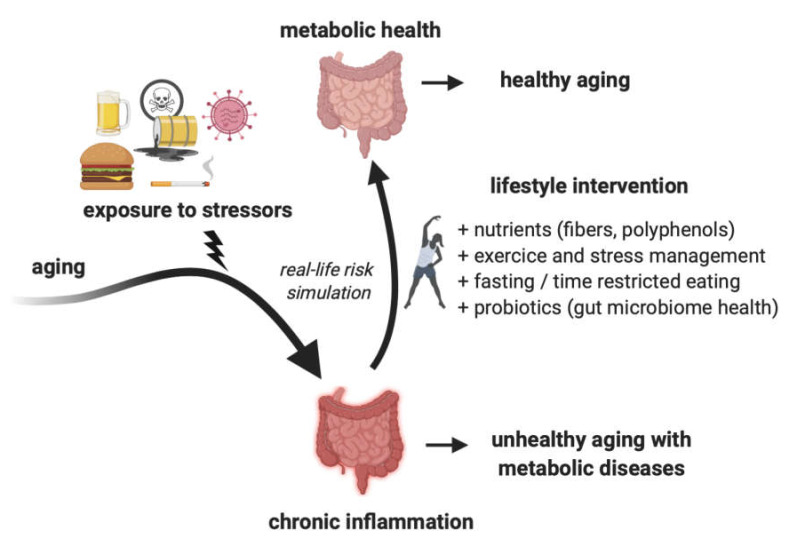
Lifestyle changes can promote healthy aging by mitigating the deleterious effects of stressors. Exposure to a variety of stressors (processed food, overfeeding, environmental toxicants, infectious agents, or drugs) during life course can promote chronic inflammation and the development of metabolic disorders. A large number of real-life risk simulation studies showed that this can be mitigated by lifestyle interventions.

Given that a large part of the global population suffers from various metabolic disorders, it is important to look for non-pharmacological ways to deal with these conditions. Targeted changes in lifestyle and especially diet can be economical tools to mitigate the development of metabolic disorders when they are at an early stage. These changes include increased fiber and polyphenol intake compared to the current western diets, but also well-structured, personalized fasting protocols, which can reduce the risk of metabolic disorders (Figure 3).

This could be implemented in various institutions by improving the nutritional quality of foods served in schools, hospitals, prisons, government buildings, or senior centers. Moving toward healthier food options in hospitals might ultimately help improve patient health and reduce healthcare costs.

Improving diet can have a positive effect on the immune response as a hallmark of RLRS. Since inflammation is associated with the acute pathological response to COVID-19 and other infectious diseases, improvement of the immune response and inflammatory markers may lead to an improved physiological resilience to disturbances by infectious agents such as viruses and bacteria, and possibly milder symptoms.
